# Multicomponent diffusion analysis reveals microstructural alterations in spinal cord of a mouse model of amyotrophic lateral sclerosis *ex vivo*

**DOI:** 10.1371/journal.pone.0231598

**Published:** 2020-04-20

**Authors:** Jin Gao, Mingchen Jiang, Richard L. Magin, Rodolfo G. Gatto, Gerardo Morfini, Andrew C. Larson, Weiguo Li

**Affiliations:** 1 Department of Electrical and Computer Engineering, University of Illinois at Chicago, Chicago, IL, United States of America; 2 Research Resource Center, University of Illinois at Chicago, Chicago, IL, United States of America; 3 Department of Physiology, Northwestern University, Chicago, IL, United States of America; 4 Department of Bioengineering, University of Illinois at Chicago, Chicago, IL, United States of America; 5 Department of Anatomy and Cell Biology, University of Illinois at Chicago, Chicago, IL, United States of America; 6 Department of Radiology, Northwestern University, Chicago, IL, United States of America; University of Florida, UNITED STATES

## Abstract

The microstructure changes associated with degeneration of spinal axons in amyotrophic lateral sclerosis (ALS) may be reflected in altered water diffusion properties, potentially detectable with diffusion-weighted (DW) MRI. Prior work revealed the classical mono-exponential model fails to precisely depict decay in DW signal at high b-values. In this study, we aim to investigate signal decay behaviors at ultra-high b-values for non-invasive assessment of spinal cord alterations in the transgenic SOD1^G93A^ mouse model of ALS. A multiexponential diffusion analysis using regularized non-negative least squares (rNNLS) algorithm was applied to a series of thirty DW MR images with b-values ranging from 0 to 858,022 s/mm^2^ on *ex vivo* spinal cords of transgenic SOD1^G93A^ and age-matched control mice. We compared the distributions of measured diffusion coefficient fractions between the groups. The measured diffusion weighted signals in log-scale showed non-linear decay behaviors with increased b-values. Faster signal decays were observed with diffusion gradients applied parallel to the long axis of the spinal cord compared to when oriented in the transverse direction. Multiexponential analysis at the lumbar level in the spinal cord identified ten subintervals. A significant decrease of diffusion coefficient fractions was found in the ranges of [1.63×10^−8^,3.70×10^−6^] mm^2^/s (*P* = 0.0002) and of [6.01×10^−6^,4.20×10^−5^] mm^2^/s (*P* = 0.0388) in SOD1^G93A^ mice. Anisotropic diffusion signals persisted at ultra-high b-value DWIs of the mouse spinal cord and multiexponential diffusion analysis offers the potential to evaluate microstructural alterations of ALS-affected spinal cord non-invasively.

## Introduction

Amyotrophic lateral sclerosis (ALS) is a progressive neurodegenerative disorder with unclear underlying etiology that involves progressive degeneration of motor neurons in the motor cortex, brain stem and spinal cord [1–3]. Degeneration of upper and lower motor neurons in ALS is associated with both sporadic and genetic alterations in various cellular pathways [2]. Current assessment of ALS is based upon a combination of clinical symptoms and examinations of upper and lower motor neurons including: electromyogram and nerve conduction studies, genetic testing, magnetic resonance spectroscopy and structural MRI [2,4]. The application of novel MRI techniques may contribute to the identification of nerve tissue changes ultimately advancing the early diagnosis, stratification and prognosis of ALS and other neurodegenerative diseases.

The microstructure of damaged nerves in ALS spinal cord tissue can potentially be reflected in altered diffusion properties amenable to detection by diffusion-weighted (DW) MRI [2,5,6]. A recent large-scale diffusion tensor MRI study demonstrated decreasing fractional anisotropy (FA) of corticospinal tracts in ALS affected patients [7]. However, multiple pathological alterations can lead to insufficient reliability in DTI measurements, including coexistence of axonal degeneration, inflammation, and demyelination [8]. Diffusion behavior in biological tissues can be complex due to tissue heterogeneity, vascularity and cellularity. Studies have revealed that, in biological systems, water proton signal attenuation due to diffusion weighting deviates from free diffusion or mono-exponential behavior at relatively high b-values (≥3000 s/mm^2^) [9–13]. Even at ultra-high b-values (up to 850,000 s/mm^2^), strong signals were found to persist in DW images (DWI) of the mouse spinal cord [14]. Variation of these strong signals acquired at ultra-high b-values can provide important information related to the pathological changes of ALS during neuronal degeneration.

The potential new information carried by DWI at ultra-high b-values and its relationship to pathological changes necessitate systematic studies on MR signal decay spanning a wide range of b-values. Various diffusion models have been explored to interpret the complicated water diffusion behavior in living tissues [15–17]. In order to interpret signal attenuation with b-values over a wide range that covers extensive high b-values, biexponential and other complex models have been applied [18–22]. While these diffusion models provide various means to capture tissue complexity, a multiexponential relaxation model, proposed by Whittall, can be applied to analyze complicated diffusion behaviors [23]. This multiexponential model assumed multiple compartments with various diffusion coefficients exist in tissues. This multiexponential model has been widely used for T_2_ relaxation analysis, and is based on a regularized non-negative least squares (rNNLS) algorithm, to identify and characterize multiple water compartments in normal and pathologic tissues, for example, multiple sclerosis [23], cartilage degeneration [24], and myelin water imaging [25]. An NNLS approach was recently applied to measure diffusion and perfusion fraction in vertebral bone marrow [26]. In addition, NNLS was used as a method to remove undesired signal contamination in diffusion compartments and for multifiber reconstruction in the presence of intravoxel orientational heterogeneity for DTI tractography [27,28]. However, no study has been reported using the NNLS approach to evaluate tissue diffusion relaxation over a broad range of high b-values in the spinal cord.

Here, we hypothesize that multiple diffusion compartments can be depicted in the mouse spinal cord by the multiexponential relaxation model. We further anticipate that ALS-induced microstructure alterations can be defined by changes in the distribution of diffusion compartmentation using multicomponent analysis. To evaluate this hypothesis, we used an rNNLS to identify potential diffusion measurement differences in the lumbar spinal cord of wild type and transgenic SOD1^G93A^ mice, a well-established ALS model. Specifically, we analyzed a series of DWIs from the spinal cords of SOD1^G93A^ mice and controls with b-values ranging from 0 to 8.5 x 10^5^ s/mm^2^ using the rNNLS method, making no prior assumptions about the number of diffusion components present.

## Materials and methods

### Sample preparation

Two groups of male mice were used for this study: A SOD1^G93A^ group (n = 7) and a wild type control group (n = 8) at early symptomatic stage (postnatal days 90–100). All experimental procedures were reviewed and approved by the Institutional Animal Care and Use Committee (IACUC) of Northwestern University (Northwestern IACUC Approval number: IS00002018) and were in accordance with the National Institute of Health Guide for the Care and Use of Laboratory Animals. Mutant SOD1^G93A^ mouse and its wild type control were purchased from the Jackson Laboratory (B6SJL-Tg (SOD1*G93A) 1Gur/J and stock No: 002726), and were housed and cared by the Center for Comparative Medicine of Northwestern University. In brief, all animals were housed under diurnal lighting conditions (12-h light/12-h dark cycles) with free access to food and water. The room was controlled to provide a relative humidity of 45 ± 5% and a temperature of 20 ± 2°C. Before the MRI scans, the mice were euthanized by CO_2_ chamber and followed by opening of the chest cavity, procedures approved by our IACUC. The spinal cords were dissected for histological analysis after MRI.

### MRI

All MRI studies were performed using a 9.4 T MRI scanner (Agilent, Santa Clara, CA), a gradient set with maximal gradient strength of 100 Gauss/cm, and a 39 mm birdcage quadrature RF coil (Rapid, Germany). A diffusion weighted stimulated echo sequence was applied with the following acquisition parameters: TR/TE = 2000/30.5 ms, mixing time = 382 ms, diffusion time (*Δ*) = 400 ms, diffusion gradient duration (*δ*) = 11 ms, slice thickness = 1.5 mm, field of view (FOV) = 36 mm × 50 mm, matrix = 64 × 96, average = 25, and 30 b-values ranging from 0 to 858,022 s/mm^2^ with a maximal diffusion gradient strength of 50 Gauss/cm. Two diffusion gradient directions were applied with one oriented parallel and the other perpendicular to the long axis of the spinal cord. T_2_ weighted images were acquired using a fast spin echo sequence with parameters: TR/TE = 1000/12 ms, echo train length = 8, matrix = 192 × 256, FOV = 36 mm × 50 mm, slice thickness = 1.5 mm, averages = 2. The bore temperature was 18–20°C measured with a thermocouple (SA Instruments, NY)

### Data processing

Image post-processing was performed in Matlab (MathWorks). Signal-noise-ratios (SNRs) were calculated from regions of interest (ROIs) manually drawn at the lumbar level in the spinal cords of both SOD1^G93A^ and wild type mice. Normalized signal intensities (I_n_) were calculated for each mouse in both groups with the following equation:

$$I_{n}=I_{m}/I_{0}$$ [1],

where I_m_ is the measured signal intensities at all b-values, and I_0_ is the measured signal intensities when the b-value was equal to zero.

The multiexponential relaxation model was used to analyze normalized signal intensities on 200 possible diffusion coefficients (D) that were logarithmically spaced over the interval of [1×10^−8^,1×10^−1^] mm^2^/s. Briefly, for a given matrix A, the normalized signal intensity of each voxel in DWIs at different b-values can be considered as a column vector ${\vec{I}}_{n}$ (Eq 2)

$${\vec{I}}_{n}=A∙\vec{W}$$

$$A=e^{-\vec{b}∙\vec{D}}$$ [2],

where $\vec{b}$ is a row vector containing the thirty b-values from 0 to 858,022 s/mm^2^, $\vec{D}$ is a column vector with 200 possible diffusion coefficients that were logarithmically spaced over the interval of [1×10^−8^,1×10^−1^] mm^2^/s and $\vec{W}$ denotes the weights of possible diffusion coefficients (or D weights). To minimize noise influence in analysis, we employed the rNNLS method (Eq 3) to find the optimized $\vec{W}$ by minimizing the “energy” in the spectrum [29],

$${Argmin}_{\vec{W}}\;\;{||A∙\vec{W}-{\vec{I}}_{n}||}_{2}+\mu ∙{\vec{W}}^{H}\vec{W},\;subject\;to\;\vec{W}\geq 0$$ [3],

where ${\vec{W}}^{H}\vec{W}$ is a regularizer and μ is the weight of the regularization term. To pick up an appropriate μ value for this study, we employed a ‘least squares-based constraints’ method that slightly regularizes the optimization function on a percentage basis of χ^2^ misfit [30]. The χ^2^ was defined as

$$\chi^{2}={\left({\vec{I}}_{n}-\hat{I}\right)}^{H}({\vec{I}}_{n}-\hat{I})/\sigma^{2}$$ [4],

where *σ*^2^ is the variance of noise, ${\vec{I}}_{n}$ and $\hat{I}$ denote the normalized acquired data and estimated data respectively. The μ was iteratively updated until Eq 5 was achieved

$${\chi^{2}}_{reg}={\chi^{2}}_{nonreg}(1+\alpha )$$ [5],

where χ^2^_reg_ and χ^2^_nonreg_ are the regularized and unregularized least squares misfit. In our study, α was set to 9% considering the relatively low SNRs [30].

The extracted D weights (or $\vec{W}$) corresponding to each diffusion coefficient were averaged in SOD1^G93A^ and wild type groups respectively. To simplify comparison of diffusion component fractions, the “crossover points”, where the values of averaged D weights (<W>) in SOD1^G93A^ and wild type groups invert, were then determined. These “crossover points” divided the total D spans into subintervals. The sum of averaged D weights (S_aDw_) for each subinterval, defined by Eq 6, was computed respectively for both SOD1^G93A^ and wild type groups.

$$S_{aDw}=\sum_{i}{<W>}_{i},\;i\;\epsilon \;subinterval$$ [6]

The difference (ΔS_aDw_) was further calculated by subtracting S_aDw_ of wild type from that of SOD1^G93A^ in each subinterval.

In addition, the sum-of-D-weights (S_Dw_) for each subinterval was computed using Eq 7 for each sample.

$$S_{Dw}=\sum_{i}W_{i},\;i\;\epsilon \;subinterval$$ [7]

Voxel-wise S_Dw_ maps were calculated in the identified subintervals for each sample. A one-dimension multiscale local polynomial transform de-noising filter was applied voxel-wise on I_n_ at different b-values prior to applying the rNNLS analysis aforementioned [31,32]. D weights were estimated for each voxel from all animals and S_Dw_ maps at individual subinterval were calculated correspondingly. The maps were overlaid with T_2_ weighted images using FSLeyes (FMRIB Analysis Group, Oxford, UK).

### Statistical analysis

A two-tailed Student *t* test was performed on S_Dw_ of each subinterval for both groups with Stata software (Stata11, Stata-Corp, College Station, TX). The statistically significant level was defined as *P* < 0.05.

## Results

**Fig 1:**
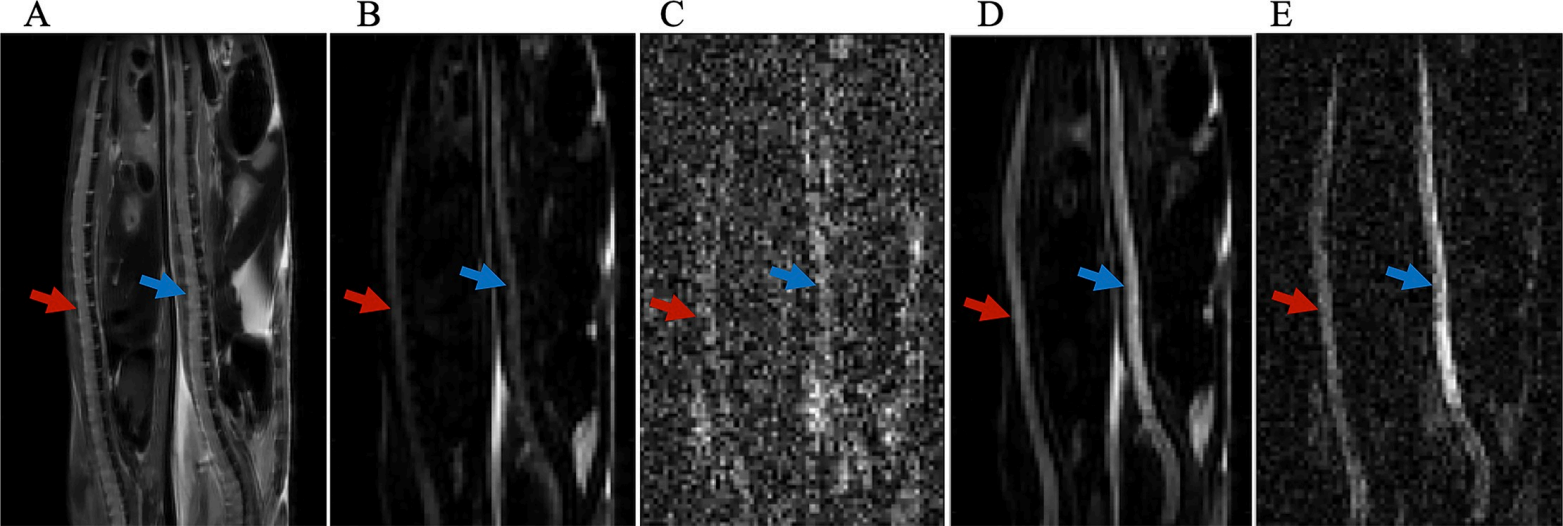
**Representative T**_**2**_**- and diffusion-weighted images of a SOD1**^**G93A**^ **mouse (red arrows) and a wild type mouse (blue arrows).** (A) T_2_-weighted anatomical image. (B) Diffusion-weighted image at b = 1.34×10^4^ s/mm^2^ with diffusion gradient direction oriented parallel to the long axis of spinal cord. (C) Diffusion-weighted image at b = 8.58×10^5^ s/mm^2^ with parallel diffusion gradient. (D) Diffusion-weighted image at b = 1.34×10^4^ s/mm^2^ with transverse diffusion gradient direction. (E) Diffusion-weighted image at b = 8.58×10^5^ s/mm^2^ with transverse diffusion gradient direction. Seven SOD1^G93A^ and eight wild type control mice were scanned to produce these representative images.

**Fig 2:**
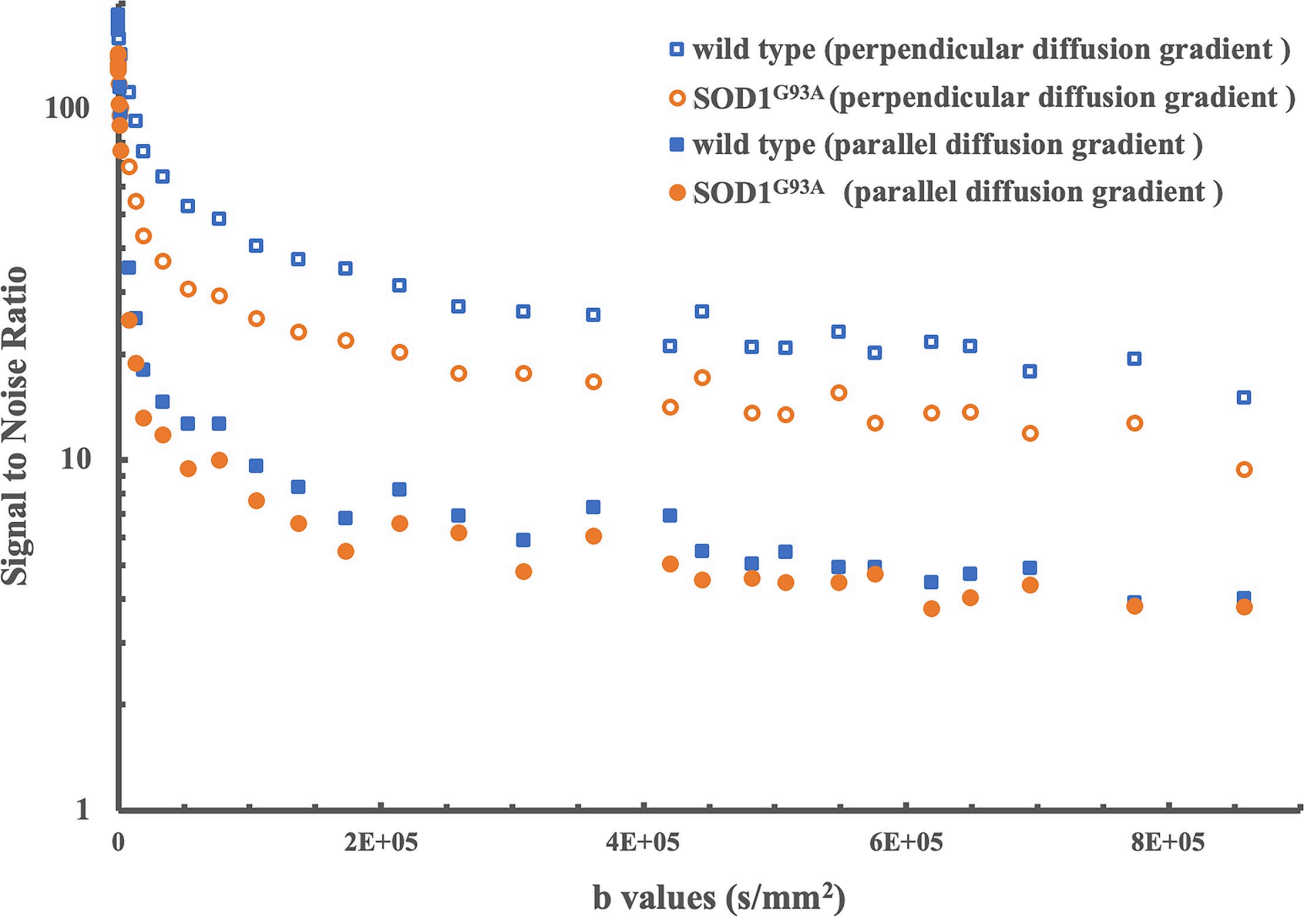
The measured SNRs from lumbar level ROIs of the representative wild type and SOD1^G93A^ mice with diffusion gradient direction parallel and perpendicular to the long axis of spinal cord. Seven SOD1^G93A^ and eight wild type control mice were scanned to produce these representative graphs.

Representative images of a SOD1^G93A^ (red arrow) and a wild type mouse (blue arrow) are shown in Fig 1. In T_2_ weighted images (Fig 1A), similar image intensities were observed in the spinal cord of the SOD1^G93A^ and wild type animals. In contrast, diffusion weighted images (DWIs) showed relatively higher signal intensities, as b-value increased, in the spinal cord of wild type mouse, compared to SOD1^G93A^ mouse (Fig 1B, 1C, 1D and 1E). The measured SNRs from an ROI obtained from lumbar level of spinal cord were lower in the SOD1^G93A^ mouse than in control mouse at all b-values (Fig 2). Relatively higher SNRs were identified in both experimental groups when diffusion gradients were applied perpendicular to the long axis of spinal cord, compared to those oriented in the parallel diffusion-weighting direction (Fig 2). In both SOD1^G93A^ and wild type mice, a non-linear behavior between the log-scaled SNRs and b-values was explicitly shown (Fig 2). Furthermore, faster SNR decays were found in the parallel diffusion direction than in the transverse direction in both groups.

**Fig 3:**
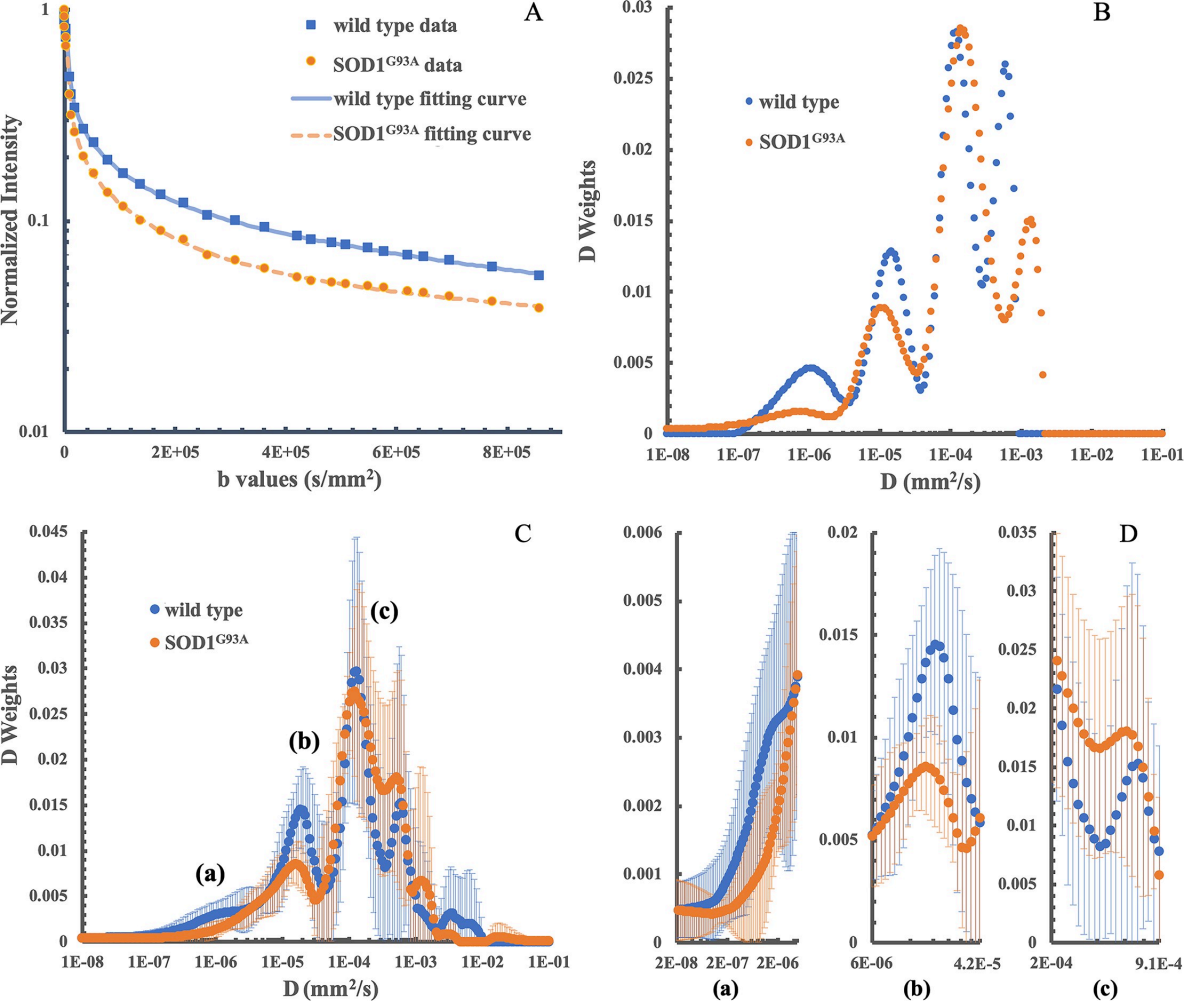
Multiexponential analysis results in a representative SOD1^G93A^ and a wild type mice and averaged D weights (<W>) for both groups (n = 7 for the SOD1^G93A^ group and n = 8 for the wild type control group). (A) Representative multiexponential fitting curves of lumbar level ROIs from a wild type and an SOD1^G93A^ mouse; (B) the corresponding distribution of D weights from multiexponential analysis. (C) The distribution of averaged D weights for both groups. (D) D weight distributions in subintervals of [1.63×10^−8^,3.70×10^−6^] mm^2^/s, [6.01×10^−6^,4.20×10^−5^] mm^2^/s and [1.66×10^−4^,8.41×10^−4^] mm^2^/s. These subintervals are labeled as (a), (b) and (c) respectively.

**Fig 4:**
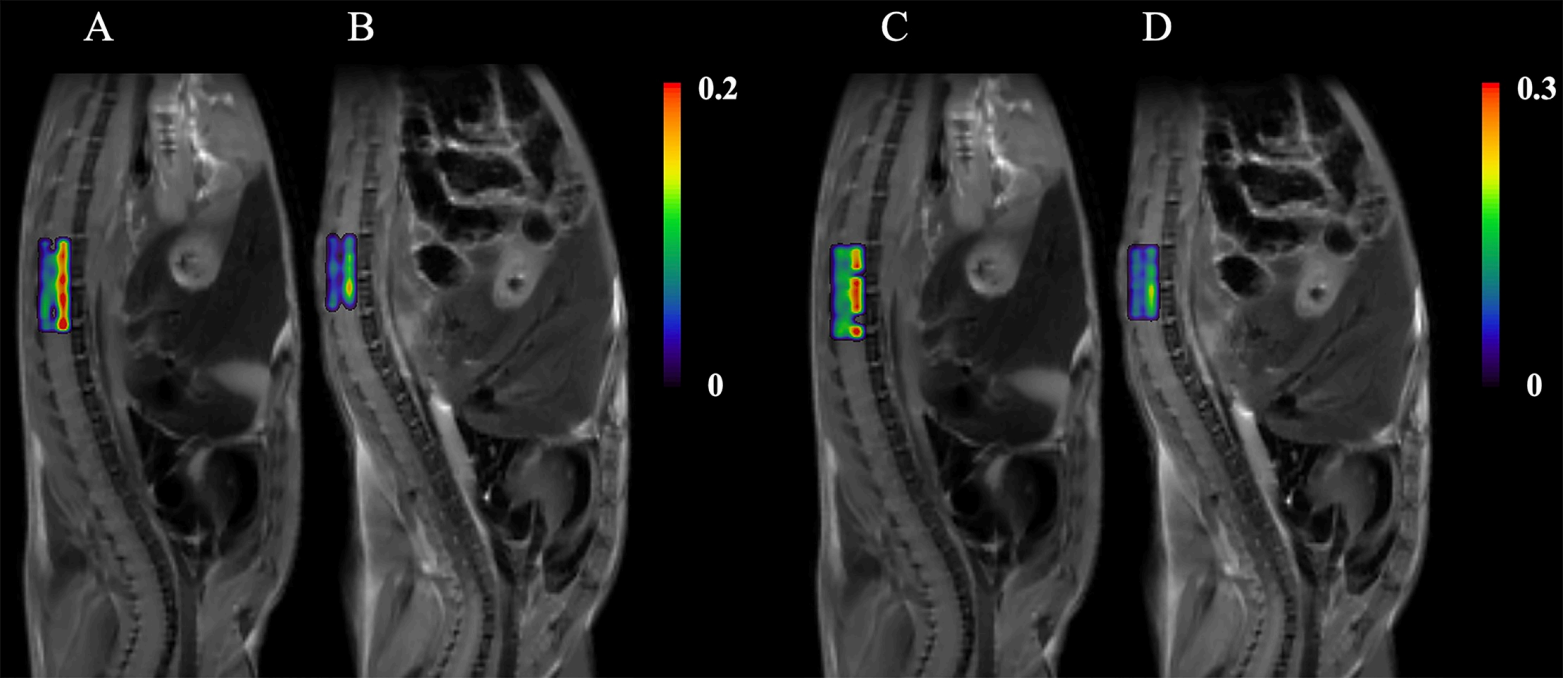
Representative S_Dw_ maps of the subinterval [1.63×10^−8^,3.70×10^−6^] mm^2^/s with color-coded overlaid on T_2_ weighted images at the lumbar level spinal cord of a wild type (A) and an SOD1^G93A^ (B) mice, and the S_Dw_ maps of the subinterval [6.01×10^−6^,4.20×10^−5^] mm^2^/s with color-coded overlaid on T_2_ weighted images at the lumbar level spinal cord of a wild type (C) and an SOD1^G93A^ (D) mice. Seven SOD1^G93A^ and eight wild type control mice were scanned to produce these representative images.

As shown in Fig 3A, a good fit was confirmed by representative rNNLS fitting curves obtained from one SOD1^G93A^ and one wild type mice when plotted on a semi-log scale from the lumbar level ROIs. The calculated weighted distributions on all 200 Ds are shown in Fig 3B. Four main peaks at 1.10 x 10^−6^ mm^2^/s, 1.47 x 10^−5^ mm^2^/s, 1.20 x 10^−4^ mm^2^/s and 6.08 x 10^−4^ mm^2^/s were observed in the control mouse. Four peaks were detected at 7.32 x 10^−7^ mm^2^/s, 1.06 x 10^−5^ mm^2^/s, 1.42 x 10^−4^ mm^2^/s and 1.37 x 10^−3^ mm^2^/s in the mutant mouse. Averaged D weights of all animals in each group provided an overall view of D weight distributions in both groups (Fig 3C). Furthermore, the largest three differences of S_aDw_, 0.0946, -0.0749, and -0.0422, were respectively located at the subintervals of [1.66×10^−4^,8.41×10^−4^] mm^2^/s, [6.01×10^−6^,4.20×10^−5^] mm^2^/s and [1.63×10^−8^,3.70×10^−6^] mm^2^/s (Table 1 and Fig 3D). A two-tailed student *t* test showed a significant decrease of S_Dw_ at the subintervals of [1.63×10^−8^,3.70×10^−6^] mm^2^/s (*P* = 0.0002) and of [6.01×10^−6^,4.20×10^−5^] mm^2^/s (*P* = 0.0388) in the mutant group. Fig 4 shows representative examples of T_2_ weighted images with color-coded overlays of the S_Dw_ maps of the identified subintervals of [1.63×10^−8^,3.70×10^−6^] mm^2^/s at the lumbar level spinal cord of a wild type (Fig 4A) and a SOD1^G93A^ mouse (Fig 4B) and of [6.01×10^−6^,4.20×10^−5^] mm^2^/s (Fig 4C, wild type; Fig 4D, SOD1^G93A^). Heterogeneous distribution patterns were observed in each of the S_Dw_ maps with hyperintense signals observed in the ventral portions of the spinal cord in the SOD1^G93A^ mouse.

**Table 1:** Sum of averaged D weights (S_aDw_) for the identified subintervals within D span.

	D (mm^2^/s)	S_aDw_ of SOD1^G93A^ n = 7	S_aDw_ of wild type n = 8	Δ S_aDw_
1	1E-8 to 1.63E-08	0.0034	0.0033	0.0001
2	1.63E-08 to 3.70E-06	0.0731	0.1153	-0.0422*
3	3.70E-06 to 6.01E-06	0.0319	0.0315	0.0004
4	6.01E-06 to 4.20E-05	0.1676	0.2425	-0.0749*
5	4.20E-05 to 1.11E-04	0.2077	0.1779	0.0298
6	1.11E-04 to 1.66E-04	0.1596	0.1679	-0.0083
7	1.66E-04 to 8.41E-04	0.3653	0.2707	0.0946
8	8.41E-04 to 9.89E-04	0.0173	0.0191	-0.0017
9	9.89E-04 to 2.05E-03	0.0513	0.0283	0.0231
10	2.05E-03 to 1.22E-02	0.0076	0.0414	-0.0338
11	1.22E-02 to 1E-01	0.0077	0	0.0077

* indicates subinterval with significant difference (*P* < 0.05)

## Discussion

MRI techniques to assess ALS progression can be beneficial in both clinical and research settings, as they may allow early diagnosis/prognosis or treatment efficacy, respectively. In this study, we found that anisotropic diffusion signals persist at ultra-high b-value in the mouse spinal cord, and that differences in signal intensity between the spinal cords of SOD1^G93A^ mice and wild type controls can be observed at an early symptomatic phase of the disease. We further demonstrated the feasibility of using multicomponent analysis to interpret DWI signal dispersion spanning an extremely large range of b-values with no *a priori* assumptions about the number of diffusion components present. Our results imply that the rNNLS multiple component analysis of a series of DWIs with b-values extended to ultra-high values can detect microstructural changes in the spinal cord associated with degeneration of motor neurons in a mouse model of ALS at early symptomatic stage.

ALS patients display extensive white matter changes involving primary and secondary motor connections indicating a network basis for the spread of pathology that can be detected by diffusion-based MRI techniques [33,34]. Diffusion neuroimaging techniques are an important assessment tool for making a precise ALS diagnosis [35,36]. Particularly, using diffusion tensor MRI, a decrease of FA within the corticospinal tract in ALS patients was consistently found to correlate with disease severity and progression in several studies [37–42]. Although MR imaging of the spinal cord is challenging, the ubiquity and non-invasive nature of MRI has supported its continued development and it now plays a leading role in ALS biomarker discovery. A better knowledge of spinal cord diffusion properties in relationship to ALS pathology could aid the assessment of ALS progression in human patients. Novel diffusion-weighted MRI techniques can be beneficial to non-invasively monitor disease progression and to evaluate treatment outcomes. Our previous study showed that strong signals persist in DWIs of mouse spinal cord, even at ultra-high b-values [14]. In this study, our results further validated the existence of complicated water diffusion behaviors in the spinal cord, as shown by the dependency of measured SNRs on the diffusion gradient directions (Fig 2) and the non-linear relationship between the measured signal intensities on log scales and corresponding b-values (Fig 3A). Additionally, we documented differences in signal intensity that arise in high b-value DWIs between the spinal cords of wild type and SOD1^G93A^ mice at disease onset (Fig 2), which can potentially reveal pathological changes of ALS. Furthermore, the high SNRs (shown in Fig 2) measured with the diffusion gradient oriented perpendicular to the long axis of spinal cord can improve the reliability of subsequent analysis on diffusion attenuation. The slower signal decay of transverse diffusion can indicate the presence of well-packed obstructions to water or other molecules (e.g., lipids in myelin) in the microstructures. Accordingly, degradation of these microstructures might be detected by transverse diffusion, even at ultra-high b-values. That being the case, DWIs with a combination of optimal diffusion gradient direction and ultra-high b-value offer the potential to probe fine structure with ultra-small diffusion coefficients.

Successful interpretation of the complex nature of tissue water diffusion and the changes that occur in disease depends on data acquisition and on the method chosen for analysis of the diffusion decay. We, in this study, utilized an rNNLS method to delineate the complex diffusion behaviors of spinal cord in a mouse model of ALS. This method allows for the separate identification of multiple diffusion components with few *a priori* assumptions of possible compartments present. As shown in Fig 3B, multiple distinct compartments with the rNNLS were extracted in this study. These diffusion components could represent water diffusion from various spinal cord white matter tracts, gray matter, and CSF filled in the central canal of the spinal cord and in the cavity between spinal cord and vertebrae, that are embodied in each DWI slice. On the averaged D weight distributions of both groups (Fig 3C), obvious differences were observed in the 7^th^, 4^th^ and 2^nd^ subintervals between mutant and wild type groups (Table 1). Among them, the 7^th^ subinterval [1.66×10^−4^,8.41×10^−4^] mm^2^/s incorporates the radial diffusivity (RD) of mouse spinal cord (approximately 4.5×10^−4^ mm^2^/s) reported in the literature [43,44]. The likely higher S_aDw_ in the mutant mouse existed when comparing to that of the control (Fig 3D), but no statistically significant differences (*P* = 0.2013) were found between the two groups in this subinterval. In the literature, RD changes in SOD1^G93A^ mice were reported inconsistently [43,45–47]. Hence, further studies on changes of D weights in this subinterval could help to explain the RD inconsistency in DTI measurements and might provide information about fine variations of microenvironment where inflammation, axonal loss, axonal injury, and demyelination coexist. We found decreased S_aDw_ in the 2^nd^ subinterval of [1.63×10^−8^,3.70×10^−6^] mm^2^/s, as shown in Fig 3C and 3D. From the Einstein’s theory for Brownian particles [48], the mean distances along the diffusion direction were in the range of [0.11,1.72] μm, which is well matched to the mean axon diameters, 0.81 μm to 1.82 μm, of various tracts of the spinal cord in B/c 57 mouse measured from histology [49]. More importantly, a significant decrease was found on S_Dw_ in SOD1^G93A^ mice at this subinterval (*P* = 0.0002). We speculate that this decrease of S_Dw_ might directly disclose the disturbance of axons in SOD1^G93A^ mice well documented in previous studies [50,51]. S_Dw_ in the 2^nd^ subinterval of [1.63×10^−8^,3.70×10^−6^] mm^2^/s can, thus, have the potential to serve as an imaging biomarker for detecting axon damage and be useful for evaluating therapeutic responses. Likewise, we found a significant decrease (*P* = 0.0388) of S_aDw_ in mutant mice at the 4^th^ subinterval of [6.01×10^−6^,4.20×10^−5^] mm^2^/s that has a mean diffusion distance range of [2.19,5.80] μm from the Einstein’s theory. The decreased S_aDw_ in this range might relate to more ‘open’ space between the remaining clusters of cellular remnants caused by destruction of neuron cell bodies and the loss of their associated axons as reported previously [50–52]. Such microstructure degradations, for example, spongiform changes of the neuropile and vacuolization of motor neurons, can lead to loss of the corresponding diffusion components and variations of other diffusion components, for example, the increase of S_aDw_ in 5^th^ subinterval (Table 1). On this account, the *D* weight distribution changes in the spinal cord of SOD1^G93A^ mice compared with the control could be due to changes of microstructure in both neurons in gray matter and axons in white matter. The *D* weights extracted from the rNNLS algorithm, hence, have the potential to interpret changes in water diffusion compartment size. Future studies are necessary to fully establish the relationship of D weight with microstructure changes at different disease stages, segmental levels of spinal cord, and the MRI acquisition parameters in both research and clinical settings.

Several limitations exist in the current study. First, relatively low spatial resolution was used in order to increase the SNR at the ultra-high b-values. One of the major challenges in spinal cord MRI arises from the small cross-sectional dimensions of the spinal cord. As a result, the large image voxel could contain multiple regions in the spinal cord e.g., gray matter, white matter tract and cerebrospinal fluid. Therefore, acquired MRI signals could have contributions from various tissues with different diffusion properties in the spinal cord, as well as from adjacent tissues such as bone, and paraspinal muscles that were also included in the imaging voxel. We also noticed that the spinal cord of wild type mice showed a greater amount of heterogeneity in the ventral portions in both high b-value diffusion weighted images (Fig 1D and 1E) and the S_Dw_ map of subinterval of [1.63×10^−8^,3.70×10^−6^] mm^2^/s and of [6.01×10^−6^,4.20×10^−5^] mm^2^/s (Fig 4). The complexity of tissue diffusion benefits the multiple components analysis with rNNLS, but on the other hand, the partial volume artifact might offset the distribution of the D weights. Therefore, while these heterogeneities have the potential to reflect microstructures and pathological changes, further studies are necessary to confirm and elucidate the source(s) of this measurement variability. Second, the subintervals were identified from the “crossover points” of average D weights in both groups. This way of analyzing the distributions of D weights is simple and straightforward, particularly when compared with other modeling methods such as the Gaussian Mixture Model [27]. However, identification of subintervals with this method is affected by the sample size of each group. Therefore, systematic studies with a large sample size and validated with other methods such as immunohistochemical and spinal cord optical coherence tomography are necessary in order to define subintervals that can precisely reflect microstructure degradation. Third, the measurements in this study were applied on ex vivo animals at the onset stage of ALS. However, progressive studies at multiple time points of disease development are necessary to establish the relationship between multiexponential diffusion analysis parameters and the early changes in the spinal cord, especially changes at the pre-symptomatic stage. One additional limitation was the high magnetic field and high gradient strength used in this study to elevate the SNR and to approach the ultra-high b-values. Further studies with clinical settings will be of value to inform the clinical translation of this method.

In conclusion, this preliminary study demonstrated the feasibility of using ultra-high b-value diffusion-weighted MRI and a multiple diffusion component analysis to evaluate microstructural alterations in the spinal cord of SOD1^G93A^ mice, a widely used ALS animal model. Further preclinical and translational studies are needed to validate the suitability of this method for monitoring disease progression and therapeutic responses in ALS and other neurodegenerative diseases.
